# Pregnancy complicated with hepatitis B virus infection and preterm birth: a retrospective cohort study

**DOI:** 10.1186/s12884-021-03978-0

**Published:** 2021-07-17

**Authors:** Shuisen Zheng, Huale Zhang, Rongxing Chen, Jianying Yan, Qing Han

**Affiliations:** Fujian Maternity and Child Health Hospital, Affiliated Hospital of Fujian Medical University, Fuzhou, 350001 China

**Keywords:** Hepatitis B virus, Preterm birth, Retrospective cohort study, Logistics regression

## Abstract

**Background:**

We aimed to investigate whether maternal chronic hepatitis B virus (HBV) infection affects preterm birth (PTB) in pregnant women.

**Methods:**

We retrospectively analyzed HBV-infected and non-infected pregnant women attending antenatal care at Fujian Maternity and Child Health Hospital, Fuzhou, China between January 1, 2016 to December 31, 2018. Participants were divided into HBV infection (*n *= 1302) and control (*n *= 12,813) groups. We compared baseline data, pregnancy and perinatal complications, and preterm delivery outcomes between groups. Performed multiple logistics regression analysis to adjust for confounding factors. Finally, we compared early PTB outcome between different HBV DNA level groups.

**Results:**

The incidence of preterm birth (gestation less than 37 weeks) was similar between the groups, early preterm birth (gestation less than 34 weeks) were significantly more among the HBV infection group than among the controls (1.6% VS. 0.8%; *P* = 0.003). After adjusting for confounding factors through logistics regression, HBV infection was found to be an independent early PTB risk factor gestation (adjusted odds ratio 1.770; 95% confidence interval [1.046–2.997]). The incidence of early PTB in < 500 group, 500 ~ 2.0 × 10e^5^ group and > 2.0 × 10e^5^ group was not statistically significant (*P* = 0.417).

**Conclusion:**

HBV infection is an independent risk factor for early PTB, and the risk did not seem to be influenced by the levels of HBV DNA. Comprehensive programs focusing on pregnant women with HBV infection would reduce the incidence of adverse outcomes.

## Introduction

Hepatitis B virus (HBV) infection is a global health problem with 2 billion people being infected worldwide, and more than 360 million are carriers of HBV. However, the morbidity varies greatly in different countries and regions. China is one of the regions where HBV infection is highly endemic [1], the infection rate of HBV in women of childbearing age may be as high as 2–8% [2, 3]. Recent researches have shown that viral hepatitis during pregnancy can increase the incidence of preterm birth (PTB), increase fetal growth, and reduce pregnancy hypertensive disorders [4]. PTB is the leading cause of perinatal morbidity and mortality. The PTB rate in mainland China can reach 7.1%, and it is on the rise [5]. Although the current studies suggest that HBV can increase the incidence of PTB [6, 7], studies on the high-risk factors related to PTB in HBV patients lacks in-depth analysis and relationship between maternal serum hepatitis B virus DNA and PTB is not yet clear. Limited evidence limits the development of specific monitoring and intervention methods. Therefore, we aimed to conduct a retrospective cohort study to analyze the relationship between HBV infection in pregnant women and PTB to provide clinical reference for pregnancy supervision and perinatal intervention, and improve the outcome of preterm infants.

## Methods

### Study design and participants

**Fig. 1:**
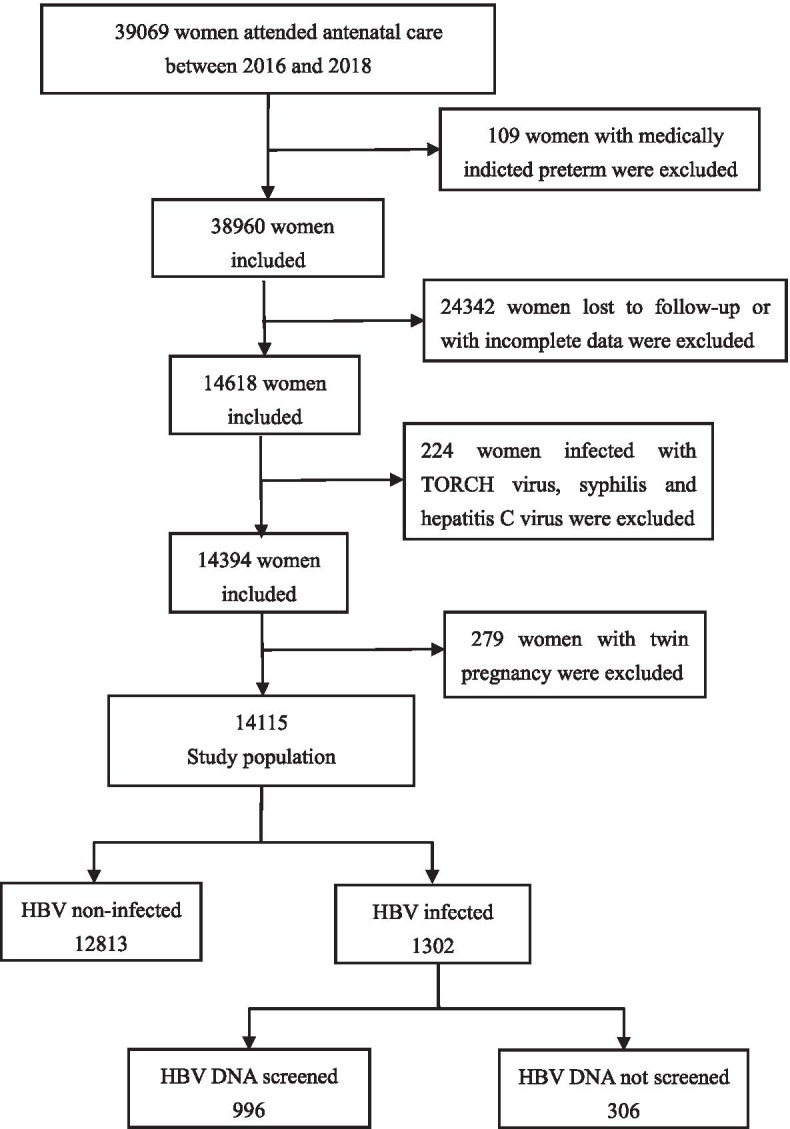
Flowchart of participants selection for the study cohort

We conducted a large population-based retrospective cohort study on pregnant women who had regular check-ups and deliveries in Fujian Maternity and Child Health Hospital from January 1, 2016 to December 31, 2018 were included in the cohort, and 14,115 pregnant women were finally included in the study (Fig. 1). According to serologic tests for hepatitis B virus in the second trimester, they were divided into the HBV infection group (1302 cases) and the control group (12,813 cases). The study was legally approved by the institutional ethics committee of Fujian Maternity and Child Health Hospital and conducted in accord with the guidelines of the Declaration of Helsinki, and the rights of all participants were protected. Consent waiver obtained from the institutional ethics committee of Fujian Maternity and Child Health Hospital.

TORCH virus includes Toxoplasma gondii, rubella virus, cytomegalovirus, herpes simplex virus and other viruses. 

Large numbers of participants lost to follow-up or have incomplete data mainly because we can’t collect up the data of serologic tests for hepatitis B. Compared the baseline situation of the women who were followed with those who were lost to follow-up or have missing data, we found that there were no significant differences in age, height, prenatal weight, the incidence of previous PTB history and the incidence of HBV infection between the two groups (Table 1). Therefore, our study is representative and credible.

**Table 1:** Compare the women who were followed with those who were lost to follow-up or have missing data

Characteristics	Women followed	Women lost to follow-up or had missing data	*P* value
Age (y)	29.55 ± 4.18	29.63 ± 4.34	0.079
Height (cm)	160.03 ± 5.17	160.12 ± 4.91	0.094
Prenatal weight (kg)	67.32 ± 8.32	67.20 ± 8.19	0.167
Previous PTB history(n(%))	252(1.8%)	450(1.8%)	0.655
HBV(n(%))	1320(9.4%)	2414(9.9%)	0.071

### Diagnostic criteria

We defined preterm births as births delivered at gestational ages less than 37 weeks and early preterm births as births delivered at gestational ages less than 34 weeks.

Chronic HBV infection was defined as either presence of HBsAg in the serum for at least 6 months or presence of HBsAg in a person who tested negative for immunoglobulin M antibodies to hepatitis B core antigen [1].

### Procedures

We collected the clinical data of the two groups of pregnant women using electronic medical records of the hospital, including: (1) General conditions: age of delivery, gestational age, pre-pregnancy body mass index ( BMI), prenatal BMI, the number of prenatal check-up, education level, previous PTB history, uterine malformation, etc.; (2) Laboratory investigations: ALT, aspartate aminotransaminase (AST), γ-glutamyl transpeptidase (GGT), lactate dehydrogenase (LDH), triglyceride (TG), cholesterol (CHOL), HBV DNA and use of antiviral therapy (4) Maternal complications: gestational hypertension, preeclampsia, chronic hypertension, abortion, placental abruption, hyperthyroidism gestational diabetes mellitus, hypertension during pregnancy, intrahepatic cholestasis of pregnancy (ICP) etc. (5) Perinatal outcome and complications: premature infants, neonatal asphyxia, small-for-gestational age infants etc. We retrospectively analyzed the differences in the baseline data, test parameters and maternal and child outcomes, and explored the correlation between HBV infection and PTB.

### Statistical analysis

The data collection and storage tools of this study were conducted using Excel (Microsoft ® Excel ® 2010), and the SPSS 25.0 statistical software was used to analyze the data in the experimental design stage to perform the normality test on the measurement data. Data that showed normal distribution are presented as mean ± standard deviation, denoted as X ± SD. The differences between the two groups were compared using the independent sample *t* test to clarify the differences between the groups. Qualitative data are expressed as frequencies and percentages, and the Pearson's chi-square test, corrected chi-square test, or Fisher’s exact test were used as appropriate. A logistic regression model was used to correct confounding factors, such as age of childbirth and BMI. The odds ratio (OR) and 95% confidence interval (CI) were calculated. *P* < 0.05 indicated that the difference was statistically significant.

## Results

### Participant characteristics

An analysis of the baseline situation between the HBV infection group and the control group found that the two groups of pregnant women had no statistically significant differences in pre-pregnancy BMI, prenatal BMI, previous PTB history, uterine malformation, and the number of prenatal check-up. The age of delivery was slightly older than that of the controls (30.22 ± 4.35 VS. 29.46 ± 4.16, *P* < 0.001), and the gestational week of delivery was slightly lesser than that in the control group (39.17 ± 2.06 VS. 39.29 ± 1.74, *P* = 0.045). There were statistically significant differences in ALT, AST, TG, ALB and CHOL levels between the two groups of pregnant women (*P* < 0.05); there was no statistically significant difference in GGT and LDH levels (*P* > 0.05) (Table 2).

**Table 2:** Maternal baseline characteristics with respect to pre-pregnancy status of hepatitis B virus infection

Characteristics	Control group	HBV group	*P* value
Age (y)	29.46 ± 4.16	30.22 ± 4.35	< 0.001
Gastational age (weeks)	39.29 ± 1.74	39.17 ± 2.06	0.045
The number of prenatal check-up	13.20 ± 2.93	13.08 ± 3.06	0.175
Pre-pregnancy BMI (kg/m^2^)	20.84 ± 2.76	20.80 ± 2.75	0.621
Prenatal BMI (kg/m^2^)	26.21 ± 3.21	26.06 ± 3.80	0.103
Education			
Primary school or below (n(%))	84(0.7%)	7(0.5%)	0.005
Junior and Senior high school (n(%))	2738(21.5%)	328(25.4%)	
College or higher (n(%))	9941(77.6%)	958(74.1%)	
Previous PTB history(n(%))	224(1.7%)	28(2.2%)	0.296
Uterine malformation (n(%))	118(0.9%)	16(1.2%)	0.275
ALT(U/L)	12.94 ± 16.81	17.20 ± 17.16	< 0.001
AST(U/L)	17.18 ± 8.51	20.68 ± 13.12	< 0.001
GGT(U/L)	14.86 ± 10.67	14.46 ± 9.41	0.196
ALB(g/L)	34.86 ± 2.77	34.47 ± 2.91	< 0.001
CHOL (mmol/L)	6.32 ± 1.18	6.03 ± 1.18	< 0.001
TG (mmol/L)	3.35 ± 1.40	3.15 ± 1.31	< 0.001
LDH (U/L)	194.06 ± 83.81	190.79 ± 72.68	0.129
TBIL (umol/L)	10.23 ± 3.10	10.38 ± 3.31	0.097

### Pregnancy outcomes complications of the study population

There was no significant difference in the incidence of premature rupture of membranes, gestational diabetes mellitus, preeclampsia, gestational hypertension, HELLP, hypertensive disorders of pregnancy, chronic hypertension, miscarriage, or placental abruption. ICP and hyperthyroidism showed statistically significant incidence between the groups (Table 3).

**Table 3:** Pregnancy outcomes, complications and neonatal outcomes of the study population

	Control group	HBV group	*P* value
PROM (n (%))	3859 (30.1)	367 (28.2)	0.147
Gestation hypertension(n(%))	198(1.5)	18(1.4)	0.637
Preeclampsia(n(%))	255(2.0)	35(2.7)	0.091
Chronic hypertension(n(%))	64(0.5)	4(0.3)	0.256
HELLP (n (%))	7(0.1)	2(0.1)	0.191
HDP(n(%))	453(3.5)	53(4.1)	0.322
GDM (n(%))	1857(14.5)	210(16.1)	0.113
ICP(n(%))	94(0.7)	32 (2.5)	< 0.001
Abortion(n(%))	56 (0.4)	10 (0.8)	0.097
Placental abruption(n(%))	189(1.5)	24(1.8)	0.299
Hyperthyroidism(n(%))	196(1.5)	7(0.5)	0.004
Preterm birth before 37 weeks	598(4.7)	75(5.9)	0.077
Preterm birth before 34 weeks	103(0.8)	21(1.6)	0.003
Neotal jaundice(n(%))	3136 (24.5)	302 (23.2)	0.305
SGA(n(%))	246 (2.1)	36 (2.8)	0.039
Admission to NICU (n(%))	1055 (8.2)	119 (9.1)	0.259
Asphyxia (n(%))	64 (0.5)	10 (0.8)	0.201
LBW (n(%))	449(3.5)	73(5.6)	< 0.001
Weight of neonates (g)			
1500 g (n(%))	94(0.7)	15(1.2)	0.002
1500–2499 g (n(%))	355(2.8)	58(4.5)	
2500–3999 g (n(%))	11,806(92.7)	1173(90.7)	
≥ 4000 g (n(%))	480(3.8)	47(3.6)	

*Abbreviations*: *GDM* Gestational diabetes mellitus, *HDP* Hypertensive disorders of pregnancy, *LBW* Low birth weight, *ICP* Intrahepatic cholestasis of pregnancy, *PROM* Premature rupture of the membrane

### Neonatal outcomes in singleton pregnancy

There were no significant differences in the incidence of neonatal asphyxia, admission to neonatal intensive care unit (NICU), and neonatal jaundice between the two groups; there were statistically significant differences in small for weight of neonates, gestational age, and low birth weight between the two groups (Table 3).

### Preterm birth outcomes

We finally included 14,115 pregnant women in our study. A total of 673 premature babies were noted, with a PTB rate of 4.8%, and 124 premature babies with a gestational age of less than 34 weeks, with an incidence rate of 0.9%. The incidence rate of PTB was 5.9% and 4.7% in the in HBV infection group and control group, respectively. The incidence of early PTB in the HBV infection group was higher than that in the control group (1.6% vs 0.8%) (Table 3).

There were some differences in the baseline data between the two groups of study subjects. Therefore, a logistic regression model was constructed to adjust the confounding factors to estimate the OR value of PTB and early PTB in women with HBV infection. After screening the confounding factors according to the results of univariate analysis and clinical significance, age, pre-pregnancy BMI, prenatal BMI, gravida, parity, number of inspections, previous PTB history, uterine malformation, abortion history, ICP, ALT, AST, TG, and LDH were included in the model (Table 3). Univariate logistic regression suggested that HBV infection status was significantly related to the risk of early PTB. After adjusting for related confounding factors (models A-C), HBV infection was an independent risk factor for early PTB (AOR = 1.796, 95% CI [1.071, 3.012]). However, regardless of single factor or multivariate logistics regression, and there was no correlation between HBV infection and premature delivery (before 37 weeks) (Table 4).

**Table 4:** Adjusted ORs for preterm birth according to baseline data

	Unadjusted	Model A	Model B	Model C
	OR (95% CI)	AOR (95% CI)	AOR (95% CI)	AOR (95% CI)
Preterm birth				
Control group	1	1	1	1
HBV group	1.250(0.976–1.600)	1.124(0.857–1.475)	1.070(0.802–1.427)	1.031(0.771–1.378)
Early preterm birth				
Control group	1	1	1	1
HBV group	2.024(1.262–3.248)	1.820(1.096–3.021)	1.770(1.048–2.991)	1.770(1.046–2.997)

Model A: adjustment was made for age, pre-pregnancy BMI, prenatal BMI, gravida, parity and number of inspections

Model B: adjustment was made for age, pre-pregnancy BMI, prenatal BMI, gravida, parity, number of inspections, previous preterm birth history, uterine malformation, abortion history and ICP

Model C: adjustment was made for the variables used in Model B and for ALT, AST, TG, and LDH

### The association between early PTB risk and the levels of HBV DNA

We screened HBV-infected pregnant women who had undergone HBV DNA quantitative testing in the first trimester, and finally included 996 pregnant women in the study. According to the DNA load of HBV, we divided them into < 500 group, 500 ~ 2.0 × 10e^5^ group and > 2.0 × 10e^5^ group, and finally found that the incidence of early PTB in the three groups was not statistically significant (Table 5). We further divided 996 pregnant women into low level HBV DNA group, high level HBV DNA without antiviral therapy and high level HBV DNA with antiviral therapy (HBV DNA > 2.0 × 10e^5^IU/ml is high level.) based on whether they were antiviral treatment during pregnancy and finally found that the incidence of early PTB in the three groups was not statistically significant (Table 5).

**Table 5:** Evaluating quantitative viral load and use of antiviral therapy

	PTB before 34 weeks (n)		*P* value
	YES	NO	
HBV DNA (IU/ml)			
< 500	11	661	0.417*
500 ~ 2.0 × 10e^5^	0	130	
> 2.0 × 10e^5^	3	191	
Wheather use antiviral therapy			
Low level HBV DNA	11	791	0.539*
High level HBV DNA without antiviral therapy	3	134	
High level HBV DNA with antiviral therapy	0	57	

^*^Fisher’s exact test was applied

## Discussion

The results of the study indicate that HBV infection is not an independent risk factor for PTB (< 37 weeks), but it significantly increases the risk of early PTB. Meanwhlie, we found that the risk did not seem to be influenced by the levels of HBV DNA.

There is still some controversy about the relationship between HBV infection and PTB in China and worldwide [8–10]. Jue Liu et al. studied 489,965 pregnant women through a national cohort study and found that HBsAg positive patients increase the risk of premature delivery. The risk of premature delivery in HBeAg negative and HBeAg-positive pregnant women with HBV infection increased by 26% and 20%, respectively. In addition, the risk of early PTB increased by 18% and 34% [11]. This is partly contradictory to our findings that HBV infection is not an independent risk factor for PTB before 37 weeks. Chen et al. reached similar conclusions to our findings; they investigated perinatal data and neonatal outcomes in 380 HBsAg positive and 428 HBsAg-negative women in Jiangsu province and found that the prevalence of PTB was relatively higher in HBsAg positive group (2.9% vs. 1.4%), but it failed to reach statistical significance (*P* = 0.140) [12]. Similarly, Jing Tan and colleagues conducted a retrospective cohort study of 21,947 singleton newborns and their mothers and found no statistically significant association between maternal HBsAg positivity and PTB (aOR 1.20, 95% CI 0.95–1.51) [13]. This may be related to the different characteristics of the study population. Jiangsu province and Fujian province are southeast coastal areas, where the prevalence of HBV infection is higher, while the PTB rate is lower than that in the southwest regions of China [5, 14], which may lead to insignificant results. In addition, the prospective study of Xu Zhuang et al. suggested that AST, GGT, and elevated bilirubin are independent risk factors for PTB, rather than HBsAg positivity [15]. In our study, after correcting ALT, AST and other indicators, we found that HBsAg positivity is an independent risk factor for early PTB, suggesting that the correlation between HBV infection and PTB is not a single abnormal liver function (ALT, AST). This indicates that the mechanism by which HBV infection causes PTB may be much more complicated.

The related mechanism of HBV infection and PTB is not clear. Some studies believe that the occurrence of PTB is closely related to the maternal–fetal interface. The rich blood supply of the maternal–fetal interface and the immune tolerance microenvironment created by the interaction of immune cells are the key to embryo implantation. When delivery is approaching, the in situ or recruited immune cells form an inflammatory reaction environment locally at the maternal–fetal interface, prompting the fetus to be delivered by the mother. Therefore, the maternal–fetal interface immune microenvironment regulates all aspects of pregnancy and childbirth, and disorder or abnormality in its balance can lead to miscarriage or PTB [16]. The accumulation of HBV-DNA in the placenta and trophoblast cells may trigger the placental inflammatory response at the maternal–fetal interface, prompting the fetus to be discharged from the mother. However, whether there is a dose–response relationship between maternal serum hepatitis B virus DNA and preterm birth is still controversial. Ioannis S. Elefsiniotis et al. found HBV-DNA positivity is significantly associated with spontaneous preterm birth [17], but they suggested that PTB risk did not seem to be influenced by the levels of HBV DNA in another paper which is similar to our findings [6]. We found that no statistically significant association between different HBV level groups in early PTB outcome. On the one hand it may be explained by low-grade inflammation response in maternal–fetal interface [18, 19]. On the other hand, Zhihua Wan et al. believe that HBV-DNA levels vary in different periods of pregnancy, and that placental inflammation may be caused by HBV-DNA in the second trimester [20]. However, what we collect is the data of HBV DNA in the first trimester which may lead to insignificant results. Therefore, it is necessary to monitor DNA levels during pregnancy to further explore the relationship between early PTB and DNA levels.

In addition, the relationship between HBV infection and other pregnancy complications and outcomes is controversial. In this study, by comparing the general baseline characteristics of the HBV infection group and the control group, we found that there was no significant difference in the incidence of GDM, PROM, gestational hypertension, preeclampsia, HELLP, and between the two groups, which is similar to the findings of Bajema et al. [21]., Reddick et al. [22], Sirilert et al. [23], and Cui et al. [24]. At the same time, the difference in the incidence of miscarriage between the two groups was not statistically significant, which contradicts the previous belief that HBV infection is an independent risk factor for miscarriage by Ai-Ming Cui et al. [24]. The reason for this difference may be attributed to the inclusion of pregnant women from 12–18 weeks and who underwent regular check-ups. Therefore, pregnant women who had miscarriages before 12 weeks were not included in the study. In addition, we also found that the two groups had statistically significant differences in intrahepatic cholestasis during pregnancy (*P* < 0.001). In the meta-analysis conducted by Jiang et al. [25], we found that HBV infection was a high-risk factor for intrahepatic cholestasis during pregnancy. Cai et al. [26] also reached a similar conclusion. As for neonatal outcomes, we found that the two groups had statistically significant differences in birth weight (weight of neonates, LBW, and SGA), which was different from the conclusions of Lao’s [27] and Connell’s [28] studies.

This study is a single-center retrospective cohort study. There are some limitations of retrospective studies. A large numbers of participants lost to follow-up or have incomplete data which may course selection bias. However, as a tertiary hospital in Southeast China, the number of patients included in the study is representative, and the results have certain reference value. Nevertheless, further prospective cohort and multicenter research is warranted to expand the research results and promote the research conclusions.

## Conclusion

In summary, pregnancy combined with HBV infection can increase the risk of early PTB. but the risk did not seem to be influenced by the levels of HBV DNA. Clinically, pregnant women with HBV infection must be closely monitored, including careful screening of high-risk women. During pregnancy, we must evaluate the condition and provide PTB-related examinations, such as fetal fibronection (fFN) testing and ultrasound measurement, as appropriate. Cervical canal length, abdominal palpation to evaluate uterine contractions, along with timely preventive cervical cerclage or vaginal progesterone administration for high-risk women help reduce the incidence of adverse outcomes.

## Data Availability

Data were anonymized, and no patient information was included to preserve confidentiality. All data used to reach the aforementioned conclusions is available from the corresponding author on reasonable request.

## Abbreviations

HBV: Hepatitis B virus
PTB: Preterm birth
BMI: Body mass index
ALT: Alanine aminotransferase
HBeAg: Hepatitis B e-antigen
AST: Aspartate aminotransaminase
GGT: γ-Glutamyl transpeptidase
LDH: Lactate dehydrogenase
TG: Triglyceride
CHOL: Cholesterol
GDM: Gestational diabetes mellitus
HDP: Hypertensive disorders of pregnancy
LBW: Low birth weight
ICP: Intrahepatic cholestasis of pregnancy
PROM: Preterm premature rupture of the membrane
fFN: Fetal fibronection
